# On Excision of Cancer of the Breast by Scissor-Cutting under Ether Spray

**Published:** 1874-11-15

**Authors:** 


### Gleanings from Our Exchanges.

### ON EXCISION OF CANCER OF THE BREAST BY SCISSOR-CUTTING UNDER ETHER SPRAY.

*From The Doctor, Oct. 1st.*

DR. Benjamin W. Richardson, F.R.S., publishes in the *Lancet*, Aug. 29, an important paper on this subject, from which we find he has himself performed operations with scissors. If this be contrary to the custom of the College of Physicians of which Dr. Richardson is a member, we none the less rejoice that he has refused to be thus trammeled, and we invite special attention to this, his last contribution to our art.

Two cases are related in detail by Dr. Richardson, the results of which he sums up as follows:

*The effect of the local anæsthesia.*—It is certain that in both these cases the local method afforded everything that could be desired in the way of anæsthesia. It saved all acute pain ; it saved the patient the dread of death during the insensibility from a general anæsthetic, and it enabled me to proceed in our task without a thought as to the immediate safety of the patient. It warranted me in recommending the operation.

*The method of cutting with scissors.*—Local anæsthesia has many disadvantages. It is more troublesome than general anæsthesia as a detail of practice, and, as it leaves the consciousness alive, it fails at times in preventing the fears of the patient. But hitherto the greatest difficulty in operating under it has been the obstacle of cutting through the hard, frozen, insensible part. The resistance to incision by the best cutting knife, and especially to dissection by the knife, is such that I have seen the most skillful surgeons troubled by it; and I have never been able to complain of the objection that had been made to the method on this ground. The difficulty is now overcome by the process of scissor-cutting, which I have here introduced. The advantage of the scissors over the scalpel will be at once proved by any one who will take a thick, firm structure—the cover of a book for example—and try to cut through it. With the best of scalpels he will be troubled ; but with scissor blades he will cut with the utmost facility, if the blades be well set. So, in cutting through the frozen animal tissue, the parts can be divided as rapidly as may be wished with the scissor-blades, with perfect accuracy of incision, and as deeply as may be desired. The cutting is also made without any downward pressure, by which pain of pressure is saved. Also in deep dissection the tissues, frozen as they are exposed, can be divided more easily than by the knife ; for the harder they are solidified, the easier they are divided by the scissor-blades. In a word, I believe that every cutting operation, in which local anæsthesia is practicable, may be performed neatly and effectively by scissor-cutting, and that a much larger number of operations may now be painlessly carried out under the local method.

*Effect of the operation on the heart in the cases related.*—No fact is more instructive in the history of the patients recorded in this paper than the beneficial effect produced on the functions of the heart by the operation. In both instances the cardiac irregularity and irritability were purely due to irregular nervous supply—to nervous irritation and consequent muscular exhaustion. The irritation might have been in part due to the mental anxiety which naturally accompanies the disease, or it might have been due to the irritation of the tumor, and have been reflex in character. Whichever view be correct, the result of the operation was curative, and, as the cases are typical of a class of phenomena of disease, the lesson they teach is extended far beyond them as individual illustrations. They show that so soon as the heart obtains rest from the persistent nervous thrill that invades it, its muscular tone returns, and its irregular motion and excitability cease. Thus, by operating early for the removal of cancer the surgeon acts as physician also, and prolongs the general life by removing the local disease. I am convinced I have seen patients suffering of cancer die from the mental and local irritation of the disease long before any development of the malady has advanced to kill by destruction of the part or organ involved.

---

Composition of Certain Kinds of Food.—Mr. J. W. Cooper read an interesting paper on this subject before the British Association for the Advancement of Science. Farinaceous foods, he said, were of the utmost importance to children and invalids, whose stomachs are too delicate to properly digest ordinary alimentary substances; and the upper and middle classes are in the habit of using with advantage considerable quantities of such preparations as arrowroot and cornflour. These substances, being in a minute state of division, break up easily and blend with water readily, and they make the food so gelatinous that the digestive juices can at once attack it. If large quantities of vegetable matter be introduced into the stomachs of delicate children, it becomes very difficult to digest; but there is nothing to prevent arrowroot, sago, and cornflour from being digested in the case of invalids and children, except in the case of children under nine months old. Excess of substances containing nitrogen has been known to produce diarrhœa in some children, as well as in grown-up persons. The nutritious value of starch foods has long been universally recognized. In India, China, Mexico, and other places, nine-tenths of the food consumed is mainly starch. In Ireland it furnishes 80 per cent., in the potato. In England our dietaries are apt to be too nitrogenous, and hence the great value and necessity for assimilative farinaceous food. A complete dietary has not yet been introduced to public notice in the form of food for adults, though Liebig did introduce a food for infants, devised upon chemical principles, to form a substitute for mother's milk, prepared from malt-flour, wheat-flour, cow's milk, bicarbonate of potash and water. Milk stands alone as a complete natural dietary for infants. The complex nature of Liebig's composition, which the author said does not appear to be much used in England, will afford some idea of the difficulties to be encountered in concocting a complete dietary; and, under these circumstances, it is fortunate, he observed, that the views of a chemist, which, if supported, were well calculated to prevent the use of most valuable and important foods for the sick chamber and nursery, have met with such decided refutation.—*Lancet*, Sept. 5,'74.

TREATMENT OF ACUTE AND CHRONIC BRONCHITIS AND ASTHMA. —Mr. Spurgin, resident medical officer to York Dispensary, employs iodide of potassium in these troublesome complaints (*British Medical Journal*, Sept. 5th, 1874). He states: "I have tried iodide of potassium in over a hundred cases with almost invariable success; in fact, with such success, that patients have expressed themselves by saying, 'It has acted like a charm'; others have said that no medicine ever had any real effect upon their complaint before. Iodide of potassium has a marked effect upon breathing, reducing the frequency of the respirations, perhaps (as I think) overcoming spasms. Almost after the first dose, patients have stated they felt the medicine touch their complaint.

"I usually prescribe it with carbonate of ammonia, and, when the cough is very troublesome, add tincture of belladonna and ipecacuanha wine. In the above complaints I rarely give anything else but the above.

"In one case of very severe broncho-pneumonia I tried iodide of potassium, with tincture of hyoscyamus and ammonia, and the respirations were quickly and astonishingly reduced from forty in a minute to less than half that number.

"In conclusion, I should add that I have purposely given a mixture containing ammonia, belladonna, ipecacuanha wine, spirit of sulphuric ether, etc., without iodide of potassium, and have not found much benefit; after which I have added iodide of potassium, and found the patient relieved almost at once."

---

IN France, as in England and elsewhere, the respective authorities and the public in general are just now taken up with the question as to the most suitable manner of disposing of their dead, as, in large cities like London and Paris, these occupy so much ground as to become an embarrassment to the living. To obviate the inconvenience of overcrowding, the different governments of France have established cemeteries outside the walls of the city, or rather, as far as possible from the centres; but the space allotted to the dead and the living, however far apart they may have been originally, soon unite, and, in course of time, they are obliged to seek other ground for the burial of the dead. This removal of the cemeteries at a distance from town is attended with great inconvenience for the proper performance of the funeral ceremonies practised in Christian countries. But, besides these considerations, there are others of equal, if not of greater importance, which ought to be taken into account: I refer to the public health. With the exception of a few of the old school, or *routiniers*, it is admitted by all hygienists that the situation of churchyards or cemeteries in the midst, or even in the vicinity of, living population, must be prejudicial to their health. It is this grave question that has led the public mind to the consideration as to the expediency of resorting to the ancient mode of disposing of the dead by incineration or cremation. But although this question is being agitated in France, or rather, in Paris, I do not think that the French will readily take to cremation, as, notwithstanding their revolutionary spirit, they are great *routiniers;* besides which, if they have no respect for anything in this world, they have great veneration for the dead; and nothing will convince them that cremation will not tend to abolish this sentiment, and the religious rites proceeding from it.—*Paris Correspondent of the British Medical Journal.*

---

THE German poet, Wieland, never lost his cheerfulness or good humor; and, but a few hours before his death, having insisted on seeing his doctor's prescription: "I see," said he "it is much the same with my life and the doctor's Latin, they are both at an end." — *Henry Crabbe Robinson's Diary.*

NELATON'S METHOD OF RESUSCITATION FROM CHLOROFORM NARCOSIS.—This method of treatment is based upon the hypothesis that death is due to cerebral anæmia, and consists in inverting the body, in order that by force of gravity the blood may be restored to the brain, while the respiration and circulation are renewed. Several striking cases of apparent death from chloroform narcosis have recently been reported in the *British Medical Journal*, in which resuscitation was accomplished by long-sustained inversion. The fact that no death from chloroform has been known to occur during labor is explained in this way: that in active labor, there can be no cerebral anæmia, inasmuch as every pain throws the blood violently to the head, producing congestion of the cerebral blood-vessels, thereby counteracting the tendency of the chloroform to produce a contrary condition.—*Boston Med. and Surg. Journal.*

---

DIGITALIS.—Dr. Gorz (*Archiv fur Experimentelle Pathologie und Pharmakologie*, 1874, p. 123) has examined, chemically and physiologically, three substances obtained by Nativelle from digitalis. These are digitalin and digitin, crystallizable and soluble in alcohol, and digitalein, amorphous and soluble in water. Digitalin and digitalein are both active, digitin inert. Gorz was able to obtain from the specimen of digitalis at his disposal only an exceedingly small quantity of digitalin, but a considerable amount of digitalein, and supposes that the dried leaves frequently contain but very little of the crystallizable principle. It seems that these facts may account for the uncertainty of the action of tincture of digitalis, which may or may not contain crystallizable digitalin, while the more reliable watery infusion is sure to contain amorphous digitalein, which is equally efficacious. Nativelle considers digitalein to be the active constituent of the officinal (not crystallized) so-called digitalin. It has generally been supposed that the diuretic effect of digitalis depends not on any specific diuretic action of the drug, but entirely upon its ability to increase the blood tension in the arterial system in general. Drs. Brunton and Power (*Centralblatt*, July 4, 1874, p. 497), however, conclude from their experiments that this is not the case, since the secretion of urine diminishes, or even ceases, when the blood pressure reaches its highest point after the injection of digitalin, and re-appears as the pressure begins to fall. They suppose that digitalis contracts the vessels of the system in general, and those of the kidney in particular, and that the latter are probably the first to relax and dilate, thus allowing a local increase of pressure in the malpighian bodies as the general pressure diminishes. They admit also the possibility of a direct action upon the secreting structure of the gland.—*Boston Med. and Surg. Journal.*

---

TREATMENT OF HÆMORRHOIDS.—Dr. William Colles (*Dublin Jour. Med. Sci.*, June, 1874), having under his care a severe case of "bleeding piles" where all former treatment, including applications of fuming nitric acid, had been of no avail, concluded to try injections of perchloride of iron. For this purpose twenty minims of the ordinary tincture were injected into each mass by means of a hypodermic syringe. The injection caused less pain than the nitric acid, and one administration sufficed to remove the hæmorrhoids completely.

---

HYPODERMIC INJECTION OF ERGOTINE IN PURPURA HÆMORRHAGICA (*The British Medical Journal*, September 5, 1874).—In a case of purpura hæmorrhagica, occurring during the progress of typhoid fever, and immediately after a severe epistaxis, two injections of one grain each of the liquid extract of ergot completely arrested the hæmorrhage from the nose, stomach, and bowels, and the patient, who was previously at the point of death from syncope, rallied and made a perfect recovery.—*Phil. Med. Times.*

NOTES OF HOSPITAL PRACTICE; CHARITY HOSPITAL, NEW YORK.—*Mucous Patches* yield in a few days to the internal administration of one-fourth of a grain of the green iodide of mercury, together with the local application of the solution of the acid nitrate of mercury. The same caustic proves very serviceable in condylomata, and is used in preference to others.

*Buboes.*—If these are the results of chancroids, any attempt to prevent their suppuration by pressure, with application of tincture of iodine, usually fails. When suppuration does take place, the dissection out of the gland by means of the handle of the scalpel is the best method to promote a speedy cure. The cavity is filled with balsam of Peru and oakum, in order to get the sore to heal from the bottom.

*Epididymitis.* — Many varities of treatment have been had recourse to, but the one that gives the best results is the tobacco-poultice. The method of using it is to make a poultice with the tobacco-leaves and apply it to the scrotum at night. In the morning, it is found that much of the pain has disappeared. The poultice serves a double service — first, that of an ordinary poultice; and, secondly, that of a depressant and nauseant.

*Phagedenic Chancroids.*—The actual cautery is used to stop the phagedenic action and serves its purpose very well.—*New York Medical Journal.*

---

THE COMIC ASPECT OF CREMATION.—The question of burning the dead is exciting much discussion in California. One paper suggests some readings on plates of funeral urns in the future: "Charles Pupker, 3¼ lbs., cremated July 9, 1879. For wife of above see third pickle bottle on next shelf. Little Tommy, burnt up September 16th, 1881. Jane Matilda Perkins, October 3d, 1883. Put up by the Alden Corpse Cremating Company. None genuine without signature."—*London Lancet.*

CROTON-CHLORAL. — Experience with croton-chloral continues to vary greatly, especially in regard to the dose. Dr. Yoe (*British Medical Journal*, March 7, 1874) does not consider it safe in any case to go beyond fifteen grains, and this amount should be reached by doses of two to five grains every hour or half hour. Dr. Durrant saw a case of severe and persistent neuralgia relieved in two days and cured in three, by one grain three times a day. In another case it utterly failed. Dr. Lewis used five grains twice a day in neuralgic dysmenorrhœa, with remarkable benefit. Dr. H. A. Allbutt used half-drachm doses every night for three weeks in insomnia. There were only three nights of calm sleep in this time. He naturally concludes that it is not of much use in sleeplessness, in which opinion, Dr. Gray (*British Medical Journal*, March 28, 1874) agrees with him, while Dr. Yoe says as a hypnotic it has no advantage over chloral. Occasionally the effect of chloral is increased by the addition of five to fifteen grains of croton-chloral.—*Boston Med. and Surg. Journal.*

---

INFLUENCE OF DIET UPON THE PROPORTION OF THE WHITE BLOOD-GLOBULES.--Wilbouchevich concludes as the result of several observations upon anæmic subjects that "a purely vegetable diet has an evident effect upon the proportion of the white blood-globules," increasing them from the normal proportion of one to six hundred red globules to one to sixty-six, and one to one hundred and thirty-eight.—*Le Progres Medical*, June 20, 1874.

---

NOVEL DRESSING FOR WOUNDS, AND FOR STOPPING BLEEDING.—M. Vigier recommends a paste made by mixing two parts of modelling clay with one of glycerine, and so making an application which will be found convenient as a dressing and preservative at the same time. — *Dublin Medical Press and Circular.*

Case of Poisoning by Digitalin.—Dr. Maguire writes a letter to the *Gaz. Hebdomadaire*, July 24, 1874, giving a case of accidental poisoning by this drug. The case was that of a woman suffering from some affection of the heart, for whom he had prescribed a granule of digitalin every evening. The patient took it upon herself to swallow a *pinch* of granules—an amount equal to one-fourth grain. In a few moments she was attacked by extreme præcordial anxiety, cold perspiration, nausea; finally, at 3 o'clock in the morning (six hours later) she vomited a small quantity of greenish, glairy matter, and experienced severe pain in the region of the stomach. Examined at 8 o'clock the next morning; the præcordial anxiety had increased, and the vomiting was recommenced as soon as any liquid was taken into the stomach. The pulse was 90 and full, the action of the heart rhythmical and strongly accentuated, the face pale, the pupils normal, no headache, but cold perspiration, general malaise, bitter taste in the mouth; finally, the patient urinated very abundantly, without pain in the renal region; she preserved perfect consciousness. A cup of coffee containing a pinch of tannic acid was immediately administered. At 11 o'clock in the morning—that is, fourteen hours after the ingestion of the poison—the patient was seized with atrocious cramps in the thighs, calves and feet; these pains returned every fifteen minutes; the pulse beat 104, full, regular, without intermissions. At 5 p.m. the pulse was 60, somewhat irregular; the face was injected, feebleness extreme, vomiting less frequent, pain diminished, tongue dry. The urine, which had been secreted very abundantly during the day, became entirely suppressed the following night. A little milk and soup were given, and were retained by the stomach. During the next day the patient's condition gradually improved; by night the pulse beat 72, rarely intermittent. In the course of the following day all symptoms improved, the bitter taste in the mouth and the extreme feebleness alone being persistent for some time.—*Philadelphia Medical Times*.

---

Tapping of the Chest.—When the fluid has been evacuated by the exhausting apparatus, the lung, in expanding, may strike against the sharp and hard canula. To prevent this M. Behier of Paris, uses a canula of soft metal to be introduced into the ordinary tube. When the pleura is emptied the soft canula bends down against the parietes of the chest, and the lung does not suffer.—*London Lancet*.

